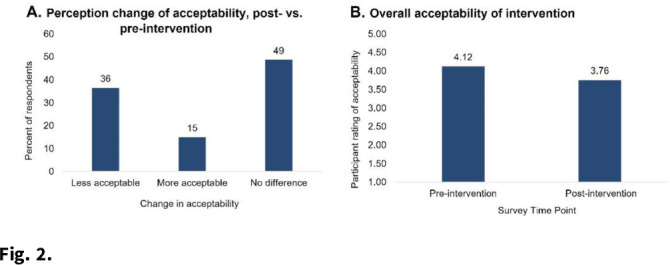# The SHIELD Study: A preliminary analysis of nasal and oral antisepsis to prevent COVID-19

**DOI:** 10.1017/ash.2022.199

**Published:** 2022-05-16

**Authors:** Julie Keating, Linda McKinley, Lin Zhao, KyungMann Kim, Thomas Friedrich, David O’Connor, Daniel Shirley, Nasia Safdar

## Abstract

**Background:** Povidone-iodine and chlorhexidine gluconate are commonly used antiseptics that have broad antiviral properties, including against SARS-CoV-2. Nasal and oral antisepsis is a possible option to reduce viral transmission; however, effectiveness data are limited. The acceptability of this method for adjunct infection control is also unknown. We are conducting a clinical randomized controlled trial (NCT04478019) to evaluate the effectiveness and feasibility of nasal and oral antisepsis to prevent COVID-19. **Methods:** Healthcare and other essential workers with in-person job duties were recruited into a 10-week clinical trial. Participation did not require in-person activities: all communication was web- or telephone-based, supplies were shipped directly to the participant, and participants self-collected specimens. Participants completed a 3-week intervention and 3-week control phases and were randomized to the timing of these phases (Fig. 1). During the 3-week intervention phase, participants applied povidone-iodine nasal swabs 2 times per day and chlorhexidine gluconate oral rinse 4 times per day following the manufacturers’ instructions for use. Participants continued all usual infection control measures (eg, face masks, eye protection, gowns, hand hygiene) as required by their workplace. To measure effectiveness against viral transmission, participants collected midturbinate nasal swabs 3 times per week to measure SARS-CoV-2 viral load. Participants also self-reported COVID-19 tests they received and why (eg, symptoms or exposure). To assess acceptability, participants completed pre- and post-surveys about their perceived and actual experience with the interventions. Participants also self-reported adverse effects due to the intervention. **Results:** As of December 3, 2021, 221 participants (148 healthcare workers and 73 non–healthcare essential workers) had enrolled. Moreover, 20 adverse effects have been reported, including skin irritation, epistaxis, and mouth discoloration; 9 participants withdrew due to side effects. Laboratory analyses are ongoing to measure effectiveness in reducing SARS-CoV-2 viral load. We performed an interim analysis of intervention acceptability. Survey responses were given on a Likert scale of 1 (not at all) to 5 (extremely). Although 36% of respondents (n = 74) reported on the postsurvey that the intervention was less acceptable than they had expected on the presurvey, the overall acceptability measure was still relatively high (3.76) (Fig. 2). In addition, 76% of respondents reported that they would use the intervention in the future (n = 56). **Conclusions:** Participant recruitment is ongoing, and data continue to be collected to analyze effectiveness and feasibility. Preliminary data suggest that participants find the nasal and oral antisepsis intervention to be an acceptable option to complement standard infection control methods to prevent COVID-19.

**Funding:** Professional Disposables International, Healthcare Division (PDIHC)

**Disclosures:** None

**Figure:**
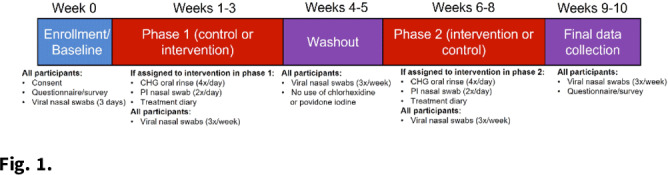

**Figure:**